# Supplementary material for: The interactome of intact mitochondria by cross-linking mass spectrometry provides evidence for coexisting respiratory supercomplexes
Source: Mol Cell Proteomics. 2017 Dec 8;17(2):216–32. doi: 10.1074/mcp.RA117.000470 (PMC5795388; doi:10.1074/mcp.RA117.000470)
Supplement: Supplemental Data [file supp_RA117.000470_133922_0_supp_23978_fzffwf.zip › spectra_annotation/mito_DR_spectra_annotation/196-1-3-1-11-1.pdf]

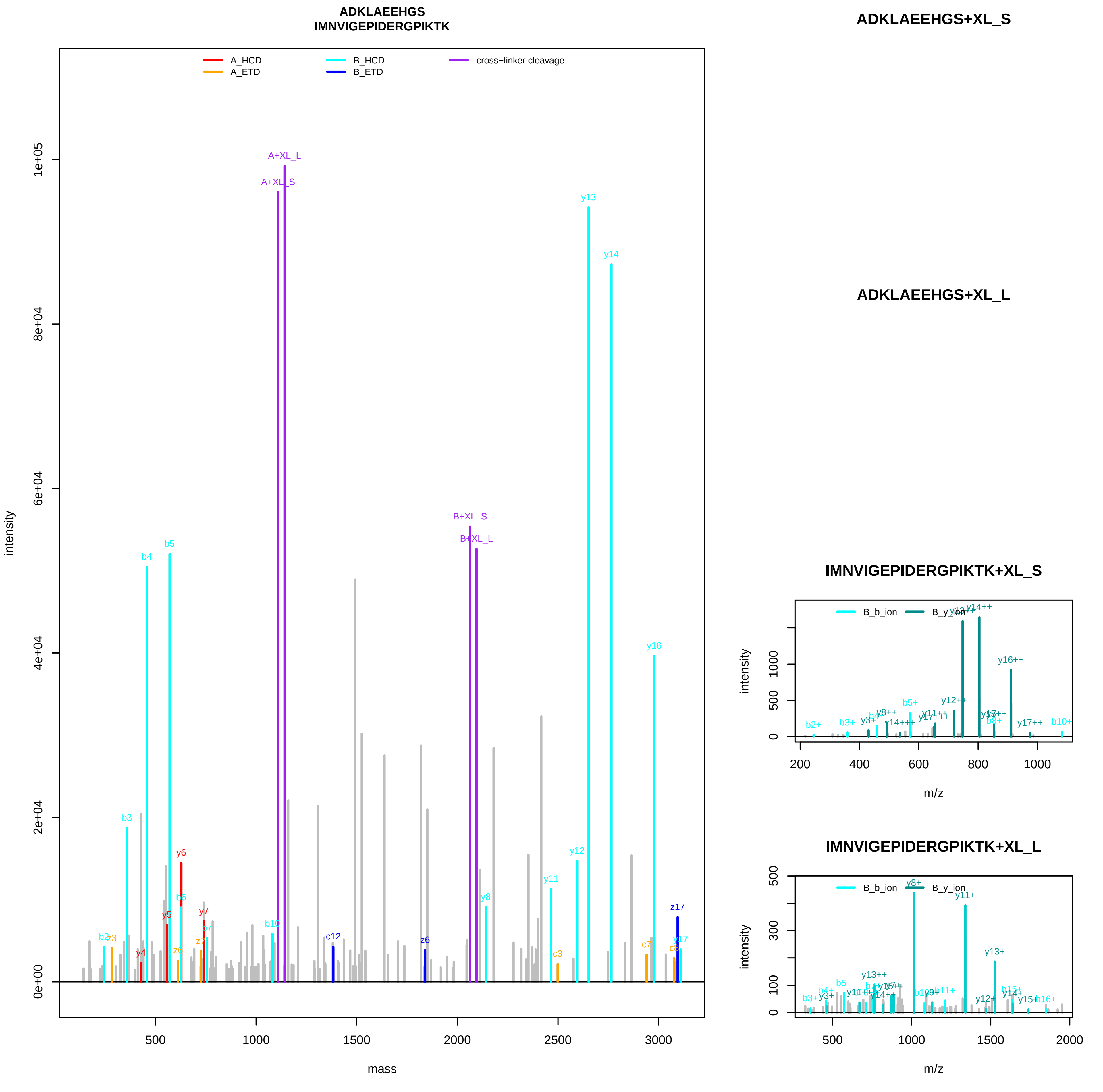
ADKLAEEHGS
IMNVIGEPIDERGPIKTK
A_HCD
A_ETD
B_HCD
B_ETD
cross−linker cleavage
intensity
mass
0e+00
2e+04
4e+04
6e+04
8e+04
1e+05
500
1000
1500
2000
2500
3000
A+XL_L
A+XL_S
B+XL_S
B+XL_L
y13
y14
y16
y12
y11
y8
b4
b5
b3
b6
b7
b2
b10
c12
z6
z17
y17
c3
c7
c8
z3
z6
z7
y4
y5
y6
y7
ADKLAEEHGS+XL_S
ADKLAEEHGS+XL_L
IMNVIGEPIDERGPIKTK+XL_S
B_b_ion
B_y_ion
intensity
m/z
0
500
1000
200
400
600
800
1000
y12++
y14++
y16++
y17++
b10+
b2+
b3+
b5+
IMNVIGEPIDERGPIKTK+XL_L
B_b_ion
B_y_ion
intensity
m/z
0
100
300
500
1000
1500
2000
y8+
y11+
y13+
y12+
y14+
y15+
b16+
b3+
y3+
b4+
b5+
b11+
